# Odontalgia. Its Pathology and Treatment

**Published:** 1853-07

**Authors:** J. P. Togg


### AMERICAN JOURNAL

OF

### DENTAL SCIENCE.

**Vol. III.** **NEW SERIES—JULY, 1853.** **NO. 4.**

### ARTICLE I.

*Odontalgia. Its Pathology and Treatment.* By J. P. TOGG, M. D.

THE simple definition of the technical term odontalgia, is to be found in our vernacular word tooth-ache. No theory whatever is implied in it. It simply means what it most emphatically is, a *pain in a tooth.* The character of this pain is Protean. It may be a slight uneasiness, it may be an intolerable anguish. It may be dull, heavy, constant like rheumatism, acute and lancinating, paroxysmal, darting, boring, throbbing. Any term of the frightful vocabulary of human suffering may be applicable to this torture. It may be cumulative, beginning moderately and increasing to intense severity, or it may at once attack the sufferer, armed in all its terrors. Yet tooth-ache is, after all, only a symptom of some lesion either in the pulp, the tooth, the jaw, the nerve, the brain, or the system at large.

The sensibility of the teeth depends entirely upon the nervous filaments distributed through that nervo-vascular mass, usually known as the pulp. Shut up in a solid box of bone, removed by several lines from the surface of the enamel, this expansion of nerves is, nevertheless, capable of perceiving very delicate impressions made upon the outside of its hard, encrusting ivory. The disagreeable sensation produced by even a few soft fragments of food, intervening between two perfectly healthy teeth, is a sufficient evidence of the delicacy of its sensibility. This natural and healthy irritability may be easily exalted to the morbid condition of irritation by impressions acting upon the pulp itself or upon the nervous system generally. Any cause, therefore, which shall unduly excite these organs may produce odontalgia. Draughts of ice-cold water, or extremely hot beverages, mechanical irritation, as blows, or hard substances forcibly pressed against them, are well known causes of tooth-ache, owing to the direct impression they make upon the nervous filaments. Any thing, indeed, which is capable of exciting pain elsewhere may excite it here. But, besides these, the teeth are subject to peculiar irritations. No other nerves are so likely to be exposed by the common accidents of disease. The regular progress of caries is to advance inwards towards the cavity, and to lay bare the pulp, by the destruction of its solid case. This having been accomplished, the sensitive centre of the organ is brought in direct contact with all those varied irritants we are constantly putting in our mouths. Like any other exposed nerve, it resents these insults, and warns us to be more cautious, by the quick, sharp, darting pain which thrills us. One of the characteristics of this form of tooth-ache is the suddenness of its invasion, and the rapidity of its subsidence, after the irritating substance is removed. To this form we may give the name, *tooth-ache of direct irritation.*

Irritation, however, may and often does run on to inflammation, so that the causes of *odontitis* or inflammation of the dental pulp will be all those accidents which have already been enumerated as sources of irritation, mechanical violence, irregularities of temperature, caries, direct contact of irritating agents, such as alimentary substances, acrid humors or hot or cold fluids touching the exposed pulp.

The pain, in this form of odontalgia, differs materially from that which we have already described. It begins, ordinarily, with a dull gnawing uneasiness in the part, accompanied sometimes by a sensation of heat. After a time, this becomes aggravated to a severe throbbing pain, which is ordinarily the precursor, here as elsewhere, of suppuration. Should the inflammation not extend beyond the pulp cavity the pain is not increased by pressure on the tooth, nor is the tooth started from its socket. The application of cold to the tooth is also followed by temporary relief.

The cause of the pain experienced in *odontitis* will be understood on the most superficial examination of the structure of a tooth. The pulp is inclosed in a hard, unyielding cavity, which everywhere adapts its walls to the soft imbedded mass. In a state of perfect health, there is no room between the pulp and the walls of its cavity. If now, inflammation sets in, it is accompanied, of course, by distension of the capillaries of the pulp, but these, meeting on all sides a hard, resisting bone, must press inwardly upon the soft, sensitive nervous filaments that are intermingled with them. The continued increase of the engorgement of the capillaries must, therefore, be accompanied by a continually increasing aggravation of the pain. Nor is this latter relieved by the occurrence of suppuration, unless the fluid gets exit through the fang, or until complete disorganization of the pulp has taken place, for the pus which is poured out only aggravates the difficulty by increasing the already intolerable pressure upon the nerves. Any excitation of the heart and arteries must also be accompanied by a marked increase in the pain. Like all other inflammations, it has exacerbations at night. These are partly due to attendant circumstances. During the day, the erect posture being maintained, the action of gravitation exerts the same influence over this, as over all other inflammatory affections. By the simple physical obstacle which it opposes to circulation it diminishes the infarction of the vessels. At night, however, the patient being in a recumbent position, the engorgement is greater, because of the removal of this salutary obstacle. The warmth of the head, also, imbedded in soft pillows, increases the disturbance.

Another point which must be observed in connection with the symptom of pain, is one which always attracts attention in other inflammatory diseases. We allude to the temperament of the patient. Every physician knows that there is great variety of susceptibility to pain in different individuals. Temperament and idiosyncrasy are both concerned in this. Nervous and sanguine persons suffer more from the same morbid change than the lymphatic and the bilious. What is the most intolerable anguish to one, is not much more than uneasiness to another. Every surgeon knows that some persons bear operations much better than others, and that, too, not from superior fortitude but actual oligæsthesia; some patients will suffer little from the gravest inflammatory disturbance, while others will be cruelly tortured by comparatively trivial organic disease.

The condition of the tooth, also, must modify the pain. One already disorganized by caries, or rendered rough by exostosis, would be more acutely painful than an organ entirely healthy.

Inflammation of the pulp may be either *acute* or *chronic*.— The first form extends to every part of the pulp and lining membrane, and terminates usually in suppuration. It is more common before than after exposure of the pulp. The second form usually results from exposure of the pulp and is not so severely painful as the acute; indeed it may last sometime without pain, but the susceptibility of the tooth to painful impressions is always increased by it.

This form of odontalgia may be called *inflammatory toothache.*

Closely allied to this is *periodontitis*, or inflammation of the investing membrane of the tooth. The same causes which produce odontitis may produce this. It is, however, more likely to be metastatic than the former variety. The tendency of fibrous membranes to metastatic disease is well known, a tendency which is very much increased by the rheumatic and gouty diatheses.

The pain, in acute periodontitis, is first dull, then acute and throbbing. It is distinguished from the disease last described by soreness and elongation of the tooth, redness and swelling of the gums and sometimes of the cheek. The tooth is sometimes so raised as to interfere with mastication. This affection often ends in alveolar abscess.

In both these forms of odontalgia, we may have all the accompaniments of inflammation, such as constipation, head-ache, dry skin, flushed cheeks, full and rapid pulse, in short, inflammatory fever.

Odontalgia may also proceed from exostosis, or the growth of bony vegetations at the base of the fang. These gradually, yet powerfully and irresistibly, pressing upon the nervous filaments must excite pain. According to Dr. Good, this pain at first is not quite so acute as in the other varieties of tooth-ache, for the pulp is not yet in a state of irritation. But by a continuance of the pressure, it is soon brought into this condition, when the pain will be as severe as on any other occasion, and far less mitigable. This disease is obscure in its symptoms and difficult of diagnosis. Often it is not detected till after the removal of the tooth.

*Fungus of the nerve*, is a cause of tooth-ache, according to Dr. Hullihen. He says, that it occupies the natural cavity of the tooth and most probably originates from a remnant of the dental blood vessels. "It always attacks teeth in which the nerve has suppurated and is of a deep red color; very soft; bleeds freely upon the slightest touch; is sometimes totally insensible, at others, highly sensitive, giving much pain of an incessant character; entirely free from any darting or throbbing sensation; and, when small and painful, is frequently mistaken for an exposed nerve. It varies in size, from that of a pin's head, to a pea and larger. It is sometimes, when small, deeply situated in a fang; but more generally protrudes, filling up a cavity formed by caries."*

So far we have considered those causes of odontalgia, which directly affect the sensitive expansion in the cavity of the tooth. We come now to investigate other and more distant causes of the same affection. Occupying an intermediate place between the local and general causes, is a condition of the nervous sys-

* American Journal of Dental Science, i, 107.

tem, alluded to by Dr. Harris*, in which the general sensibility is so exalted that ordinary impressions, which, in a state of health, are unattended by any uneasiness, may be productive of severe agony. Analogous affections of other organs are constantly met with in the practice of medicine. In many forms of fever, the cutaneous surface becomes so exceedingly sensitive as to be intolerant of the slightest touch. This is but a lower degree of the same morbid change which produces those severe aching pains in the muscles accompanying fever.

*Neuralgic tooth-ache* is an affection which may proceed from a vast variety of morbid conditions. Any disease which is capable of producing neuralgia elsewhere may bring it about in the teeth. To enter into the investigation of neuralgic odontalgia, would lead us into the consideration of all the various changes which may induce neuralgia, and would extend this little article to a most unwarrantable length. We can, therefore, glance at but a few of these morbid alterations.

We may have intrinsic or extrinsic affections, capable of producing neuralgic tooth-ache. Among the first we may enumerate *neuritis* or inflammation of the nerve, either in its substance or its neurilemma. The morbid change referred to by Cotunnius and others, namely, a deposit of a gelatinous character under the neurilemma, is probably a result of inflammation. So, too, the thickening of the neurilemma, observed by Cirillo and Earle would seem to be referable to the same cause.

A more common condition is simple engorgement of the vessels of the nerve, an affection which is often only the first stage of inflammation. This engorgement has been frequently discovered, especially in cases of sciatica, and may have been present before death, when no traces of it are found on a post-mortem examination. The experiments of Broussais and Goupil are conclusive on this point; they having found that rabbits, in which intense peritonitis existed during life, as seen by openings in their bellies, exhibited often no traces of this engorgement after death.

* Principles and Practice of Dental Surgery, 5th ed., p. 337.

Venous congestion is still another cause of this intense pain. This has often been observed about superficial nerves affected with neuralgia. The fact that most neuralgic affections are relieved by heat and aggravated by cold gives additional probability to this hypothesis of venous congestion acting as a cause to produce neuralgia. We might further cite the intimate relations between neuralgia and intermittent fever, a disease marked by intense and frequently recurring paroxysms of venous congestion.

Any pressure of the nerve along its course may be productive of the same affection. An exostosis of the walls of the infra-orbital or inferior dental canal, an aneurism of one of the accompanying arteries, a foreign body pressing upon the nerve, or a tumor affecting it at any part of its course may produce neuralgic tooth-ache. The teeth may be and often are associated in the general anguish which involves the whole face, during an attack of facial neuralgia or *tic douloureux.* This may depend upon any of the above enumerated causes. In a case of this disease, occurring at the Richmond Hospital in Dublin, the Gasserian ganglion was fibro-cartilaginous and as large as a nutmeg. Or the disease may be central and depend upon some disorder of the encephalic mass, affecting the fifth nerve at its very origin.

It must not be forgotten, however, that this general neuralgia of the face may be caused by the irritation of a diseased tooth. The phenomena of the *transferrence* and *radiation* of impressions by nervous centres have not been sufficiently studied. That they possess these properties is well known. By radiation of impressions is meant that power of the centres by which they disperse an impression made at one point, over a wide circuit in its neighborhood. Thus, to cite a case directly in point, a diseased tooth, irritating a single ramuscule of the inferior dental nerve, may arouse sufficient central disturbance to be reflected over the whole face and head, affecting every branch of the great trifacial trunk.

Transferrence of morbid impressions is analogous to radiation, but much more remarkable. In consequence of this property of nervous centres, an impression produced at one point is felt at a remote one. Disease of the hip joint, is a familiar example. This, as all surgeons know, is very often first revealed by pain in the knee. Mr. Lawrence's case, is a very striking illustration of this law of nervous centres, for in that, neuralgia of the *thumb* was caused by the pressure of a *pivot tooth on the nerve of an old fang.*

The teeth may also suffer among other organs, those strange, wandering pains which accompany and which sometimes constitute as far as symptoms can constitute a disease, the *febres larvatæ* or masked intermittents, so common in malarious districts. These may be easily recognized by the judicious practitioner who is watching for symptoms of this form of disease, but will be certainly overlooked by the careless observer who does not think of anything below the surface. As a general thing, universal *malaise* accompanies this disturbance, though but a single nerve may feel the severity of the pain; and the watchful physician can assuredly detect the characteristic element of periodicity. Sometimes, however, this is very obscure, and hardly to be recognized by the closest scrutiny, or eliminated by the most careful inquiry. As the disease advances, the periodical element gradually retires, and the neuralgic becomes unduly prominent. An analogous affection is the hepatalgia of malarious districts, so generally misunderstood, so often confounded with chronic hepatitis, so commonly mismanaged with large doses of calomel. This disease is almost always more or less regularly intermittent at first, though it finally becomes constant, or irregularly paroxysmal. In both affections, at this stage, the history of the case forms our most valuable diagnostic.

*Neuralgic tooth-ache*, however occasioned, is characterized by very much the same train of symptoms. Dr. Wood, in speaking of it, says: "The pain is usually of the acute character, sometimes mild in its beginning, gradually increasing in intensity, and as gradually declining; but usually very irregular, at one time moderate, at another slow, and occasionally darting with excruciating violence through the dental arches, not unfrequently it assumes the regular intermittent form.

Instead of pain, strictly speaking, the sensation is sometimes of that kind which is indicated when we say that the teeth are on edge, and is apt to be excited by certain harsh sounds, such as that produced in the filing of saw-teeth, by mental inquietude, and by the contact of acids or other irritant substances. Neuralgic tooth-ache sometimes persists, with intervals of exemption, for a great length of time. The diagnosis is occasionally difficult. When, however, it occurs in sound teeth, it is paroxysmal in its character, is attended with little or no swelling of the external parts, occupies a considerable portion of the jaw, and especially when it alternates or is associated with pain of the same character in other parts of the face, there can be very little doubt as to its real nature."

*Sympathetic tooth-ache* cannot, by any known means of diagnosis in symptomatology, be distinguished from the last variety. Its history alone can throw any light upon it.

The nervous connections of the teeth are so numerous and so extensive, that we need not be surprised at the wide range of their sympathies. The fifth nerve supplying the whole face and all the organs of the senses brings them in relation with the entire head. But besides this, they have special connections with many of the more important organs of the head. Meckel's ganglion and its branches, forming a sort of sympathetic centre for the entire head, is directly connected with the superior maxillary nerve, just as it is about passing into the infra-orbital canal for the supply of the upper teeth. With the ear they are still more intimately connected though the otic ganglion which rests upon the inferior maxillary nerve, at its exit from the foramen ovale. This union will explain the extreme frequency of ear-ache in children at the period of second dentition, and the tooth-ache which sometimes attends it. The same ganglion, by means of the tympanic plexus which is so directly connected with it, brings them in relation with the glosso pharyngeal and the pneumo-gastric nerves and so establishes a sympathy between these organs and the whole upper portion of the alimentary canal and with the lungs. Through the sympathetic system these connections are indefinitely extended.

With the spinal cord they have not only the union, through the origin of the fifth nerve, with the spinal bulb, but also through the numerous filaments of the sympathetic which are connected with the spinal nerves. Thus we find that, through this intricate web of sensory filaments, the teeth are directly or indirectly united with every organ in the system.

During certain conditions of the system, therefore, it is not surprising that these sympathies should be roused to undue activity. Many diseases have the power of inordinately exciting nerves remote from the organ affected. The various disturbances of the alimentary canal are remarkable for this peculiarity. The anatomical connection between the stomach and bowels and the teeth, already glanced at, sufficiently prepares us to expect tooth-ache as an occasional accompaniment of disorder of the apparatus of alimentation.

*Pregnancy* is another state which is very liable to excite sympathetic tooth-ache. This is indeed but a part of those numerous remote disturbances which are caused by the peculiar condition of the uterus at this interesting period. All the organs of the abdomen are involved in that intricate net of nervous filaments which are ultimately distributed to the uterus. The two great cords of the sympathetic which descend from the solar plexus and semilunar ganglion, on both sides of the aorta, first unite over that vessel in a plexus which weaves into itself all the various threads of which these cords and the stray fibres which surround them are composed. Having thus knit up the life of the whole abdomen in one intricate interlacement of fibrillæ, the nervous mass then divides into its two hypogastric nerves and their plexuses. These proceed directly to the ganglia of the uterus, sending off great numbers of nervous twigs to all the neighboring organs, and wrapping all the pelvic viscera in their labyrinthine threads. In this manner is the great organ of reproduction brought into relation with the entire frame. During pregnancy, as has been clearly demonstrated by Dr. Lee, the nervous system undergoes the same change to which all the other structures of this organ are subjected. They increase in size, and this increase extends even to the cords which descend from the semilunar ganglia. There are, therefore, both anatomical and physiological reasons for the extensive range of sympathies possessed by the pregnant uterus. The close contact of the semilunar ganglion with the stomach, and the relations between that organ and the teeth, explain how these may be involved.

*Rheumatism*, with its usual wandering character, may attack the fibrous structures of the teeth. The diagnosis will be made by a careful study of the diathesis, and a thorough investigation of the attendant and antecedent circumstances of the case.

*Gout*, affecting every fibrous tissue in the body, does not neglect the teeth. Dr. Harris, in a note to the chapter on tooth-ache, in his Principles and Practice of Dental Surgery, has an extremely interesting case, which we need not apologise for quoting:

"The subject, Mr. W., a resident of Baltimore, about forty years of age, had been subject to attacks of this most excruciatingly painful affection for more than fifteen years. The paroxysms, for some four or five years previous to the above mentioned time, had occurred at intervals of from three to six months, and from ten to twelve days before each attack, during this period he suffered from pain in the first right superior molar tooth, which was slightly affected with caries in the centre of the grinding surface, but it had not yet penetrated to the pulp cavity. The pain at first was not severe, but it gradually increased in intensity, and assumed a peculiar boring, and at times, a grinding and lancinating character. His attacks of gout were confined to the joint of the big toe of his right foot, and as soon as the pain commenced here it subsided in his tooth. But when the paroxysm of gout began to pass off, it again commenced in the tooth, where it continued for about two weeks. At the request of Mr. W. we removed the diseased portion of the tooth and filled the cavity. The operation for about three months promised to be successful, but about ten days previous to the next paroxysm of gout, the tooth began to ache, the pain subsiding with the occurrence of the paroxysm, but commencing again, when it passed off, and continued as it had formerly done for about two weeks. His suffering from the pain in the tooth, during these periods, was so great, that he determined to have it extracted. What the result of its removal has been, we have not yet ascertained."

The *treatment* of tooth-ache must necessarily be as varied as the disease. To talk of administering one remedy for such multiform disease is a manifest fallacy.

The tooth-ache of direct irritation has manifestly two indications, first to remove the cause, secondly to allay the pain.— The latter indication is accomplished in various ways. Opiates and other narcotics and the anæsthetics may be used to deaden the sensation. Or this may be accomplished in another way, by so vehemently stimulating the exposed nerve as to exhaust its sensibility. The essential oils, of cloves, of cinnamon, of cajeput are used for this purpose. Intermediate between the two, is kreasote, which possesses both stimulant and anodyne powers. Combined with morphia in such proportions as will form a thin paste, and applied on a small pledget of lint or cotton, it usually acts very agreeably. Dr. Harris prescribes the following formulæ:

| | | | | | |
|---|---|---|---|---|---|
| ℞ | Æther. sulphuric. | ℥ j. | ℞ | Ætheris sulphuric. | ℥ j. |
| | Pulv. camphoræ, | ʒ j. | | Kreasoti, | ʒ ss. |
| | Pulv. aluminis, | ʒ j. | | Ext. gall. Alep., | ʒ j. |
| | Morphiæ sulphat. | ʒ j. m. | | Pulv. G. camph., | ʒ ss. m. |

These are to be applied on raw cotton, after having first cleansed and thoroughly dried the offending part, and renewed as often as may be necessary. Relief is soon obtained, but of course it is not permanent. A thick solution of *gutta percha* in chloroform is another favorite remedy. It has the double advantage of allaying the pain by its anæsthetic properties, and of subsequently forming a thin but impervious coating which sheathes the nerve and protects it for a while from the irritating substances about it.

Permanent relief, however, can only be afforded by extraction of the tooth or destruction of the nerve, as there is no other way of fulfilling the indication of removing the cause. Of extraction it is not necessary to speak in this place. Destruction of the nerve may be accomplished by thrusting a sharp stilet down through the root of the tooth and by thus mechanically breaking up the structure of the nerve, or by escharotics. The most powerful of the latter is the actual cautery. A favorite mode of domestic application of this remedy is heating a small knitting needle to high redness and plunging it down into the pulp till its destruction is effected. The only caution to be observed is to introduce it so far into the fang as to destroy the entire nerve. *Potassa fusa* may be used, made into a paste with absolute alcohol, and applied to the exposed pulp. Great care should be taken not to allow this agent to come in contact with the surrounding soft parts, or much pain and troublesome inflammation and ulceration will follow. The combination of a small quantity of pure morphia with this agent will somewhat abate the pain of its operation. *Chloride of zinc* is another very powerful escharotic which must also be used with extreme caution. The most common of all these potential cauteries, however, is arsenious acid. Dr. Foster of New York, uses this in combination with morphiæ sulphas, in the proportion of two parts of the caustic to eight of the narcotic. It is applied directly over the nerve, on a small pellet moistened with kreasote, and covered with a cap, so as to avoid pressure. The subsequent treatment for the preservation of the bony shell does not come under our consideration.

In *inflammatory tooth-ache*, the dentist has to consider which of these courses he will take, whether he will extract the tooth, destroy the nerve, or attack the inflammation with antiphlogistics. "The propriety or impropriety of extraction will be determined by the amount of pain, the progress made by the inflammation, the condition of the parts with which the tooth is immediately connected, the effect of the local disturbance upon the general system, the situation and importance of the tooth, and the extent of structural alteration which has taken place in the crown. If the retention of it on account of its location, or the loss of several other teeth, is of great importance to the patient, and the circumstances of the case justify a well grounded belief that it can be preserved and rendered useful, without acting as a morbid irritant, the operation, if possible, should be avoided. In this case, supposing the inflammation to have proceeded too far to be arrested, the pulp may be destroyed and the tooth treated in the manner elsewhere described, as it would be useless to procrastinate the suffering of the patient by instituting other treatment in the vain hope of avoiding an alternative, which, after all, may not enable the dentist to secure the permanent preservation of the organ. Indeed, after the lining membrane has become exposed, this is the only method of procedure, in any stage of the inflammation, which, with a view to the preservation of the tooth, holds out any prospect of success."

If the tooth is not eroded, or if the inflammation is not caused by the direct application of irritants to the exposed pulp, the antiphlogistic treatment may succeed. It is possible that, in an intense inflammation accompanied by high sthenic febrile excitement, venesection might be desirable. As a general thing, however, the application of leeches to the gum, in the neighborhood of the diseased tooth, is the only way in which it is necessary to abstract blood in this painful little affection. Saline cathartics, and rigid abstinence constitute the rest of the treatment.

Suppuration having occurred, the treatment must be modified. This condition may be detected, according to Dr. Hullihen, by the following signs. "At first the tooth is painful only when hot or cold fluids pass over it, and may continue in this situation for some time. At length a steady gnawing pain supervenes, and then the tooth begins to get sore and appear a little loose and longer than the rest. About this period the pain assumes a very different character, darting from the tooth along the courses of the nerve, to the temple, ear, and side of the head, and to the teeth of both jaws on that side. Within twenty-four hours the face begins to swell, and the pain changes to one of a constant throbbing description which indicate that an alveolar abscess is forming. When a tooth in the manner described, becomes painful, the nerve is about suppurating; when it appears longer than the rest, loose, and is very sore, the nerve has suppurated and the pus is beginning to ooze out at the extremity of the fang where the vessels enter, producing much pressure, hence the sharp darting paroxysm. When the cheek begins to swell, the matter is effusing between the alveolus and its lining membrane, and occasions the throbbing pain which always accompanies the formation of an alveolar abscess. The true abscess of the antrum maxillare, originates in like manner, from the same cause, when the fangs are very near or penetrate into that cavity." The treatment recommended by Dr. Hullihen is the trepanning or drilling of the tooth so as to evacuate the pus.

*Periodontitis* is treated very much in the same way as inflammation of the pulp. Besides the ordinary antiphlogistic treatment, counter-irritation to the neighboring cheek may be of service. Narcotic agents combined with rubefacients have often been found to be beneficial. A deep incision into the gum has been recommended. Should alveolar abscess be threatened, extraction will be necessary. Should it be an incisor or cuspidatus which is involved, it should be removed only as a last resort. In chronic inflammation the necessity for extraction is more urgent.

*Fungus of the nerve* must be destroyed by the actual cautery. The pain may, however, be relieved by making the morbid growth bleed freely. Dr. Hullihen narrates a singular case of hemorrhage occurring monthly from fungi in a young lady's teeth. Destruction of the growth and plugging the teeth did not relieve. At last it was decided to be a case of *vicarious menstruation* and as soon as the catamenia were regulated by constitutional treatment, the local disorder vanished.

*Neuralgic tooth-ache*, originating, as it does, from a great variety of causes, demands a corresponding variety of treatment. Unfortunately, neuralgia still continues to be one of the *opprobria medicorum*, but we have of late been approximating something like a rational treatment of this obstinate and agonizing distemper.

Thus, if the disease be caused by a general exaltation of nervous impressibility, the indication is manifestly to subdue that irritability. If it be purely functional, the narcotics will accomplish this. If, however, it depends, as it may, upon loss of balance between the nervous and vascular systems, that balance must be restored, either by tonics, in one state of the system, or by sedatives, in another. Arsenic and iron are favorite remedies of the former class, aconite and the antiphlogistic regimen of the latter. Should the affection proceed from disturbance of the nervous centres, they must be attended to.

When neuralgic odontalgia depends upon neuritis of the fifth pair or its dental branches, local antiphlogistic treatment along the course of the inflamed nerve, must be adopted. In simple irritation of a nervous trunk, chloroform applied to the surface nearest the nerve, and over its course, will often prove eminently successful. If there be any tumor pressing upon the nerve, it must be removed.

Sometimes the disease continues so obstinate, that even when purely local it resists all the remedies we apply. In this case, division of the nerve has been suggested. If the inferior dental nerve be involved, M. Roux recommends trephining the lower jaw, so as to reach the nerve, which is easily recognized by its color and direction. Having exposed it, he then severs it and passes a stick of potassa fusa up and down the canal so as to completely destroy it. If the superior maxillary nerve be affected, an incision is made upon the cheek and the caustic introduced as deeply as possible along the infra-orbital canal. This operation is resorted to in those maddening neuralgias of the face which associate the teeth in their general anguish.—When the teeth thus make part and parcel of a number of suffering organs, the treatment must necessarily be general to the affected nerve. The main disturbance being quieted, all the subordinate pains are allayed. As facial neuralgia, however, may depend upon the irritation of a diseased tooth, it is necessary to examine the case minutely, and to extract immediately, if necessary. Here, however, caution is to be observed. Pain may be inflicted unnecessarily; sound teeth may be drawn without at all benefiting the patient, and on the other hand a useless organ may be suffered to remain in the jaw, a perpetual source of irritation, owing to a timid or procrastinating policy.

When tooth-ache depends upon that general neuralgic condition which accompanies malarious poisoning, quinine, arsenic and iron are the proper remedies.

In treating sympathetic tooth-ache, the first point to be determined is the nature and seat of the primary disturbance. If this can be reached and overcome, it is manifestly our duty to do it. If not, the determination of the source of irritation will have, at least one good effect; it will prevent the dentist from unnecessarily wasting his patient's teeth by profitless extraction. It would occupy us too long to attempt to trace the various morbid conditions of different parts of the system and the many modes of treating them, and then we could not succeed in giving a clear idea of all the circumstances on which this painful affection may depend. The investigation of the cause of the disease, and the selection of the appropriate remedies must be left to the judgment of the practitioner and that in each individual case.

In *rheumatic tooth-ache* the treatment will be leveled at the general disease, while the local disturbance must not be neglected. Colchicum, calomel and James' powders, guaiacum, nitrate of potash and opium at night, followed by a cathartic in the morning, will answer the first indications, and plasters of opium and belladonna the second.

In *gouty tooth-ache* the treatment must also be addressed to the main disorder.

In *tooth-ache from exostosis*, the remedy is extraction. In some cases, however, Dr. Good's mercurial treatment may succeed, for we may have syphilitic exostosis here as well as any where else. The existence of the exostosis, however, must be first established, for pure neuralgia is usually aggravated by mercury.

